# Supplementary material for: Peripheral Blood DNA Methylation Profiles Do Not Predict Endoscopic Post-Operative Recurrence in Crohn’s Disease Patients
Source: Int J Mol Sci. 2022 Sep 9;23(18):10467. doi: 10.3390/ijms231810467 (PMC9503775; doi:10.3390/ijms231810467)
Supplement: Supplementary file 1 [file ijms-23-10467-s001.zip › ijms-1871254-supplementary.pdf]

Supplementary tables

Table S1. Modified Rutgeerts' score[62].

**i0**	No lesions in the distal ileum
**i1**	<5 aphthous lesions in the distal ileum
**i2a**	Lesions confined to the ileocolonic anastomosis (including anastomotic stenosis)
**i2b**	≥5 aphthous lesions or larger lesions, with normal mucosa in-between, in the neoterminal ileum (with or without anastomotic line)
**i3**	Diffuse aphthous ileitis with diffusely inflamed mucosa
**i4**	Large ulcers with diffuse mucosal inflammation or nodules or stenosis in the neoterminal ileum

Supplementary figures

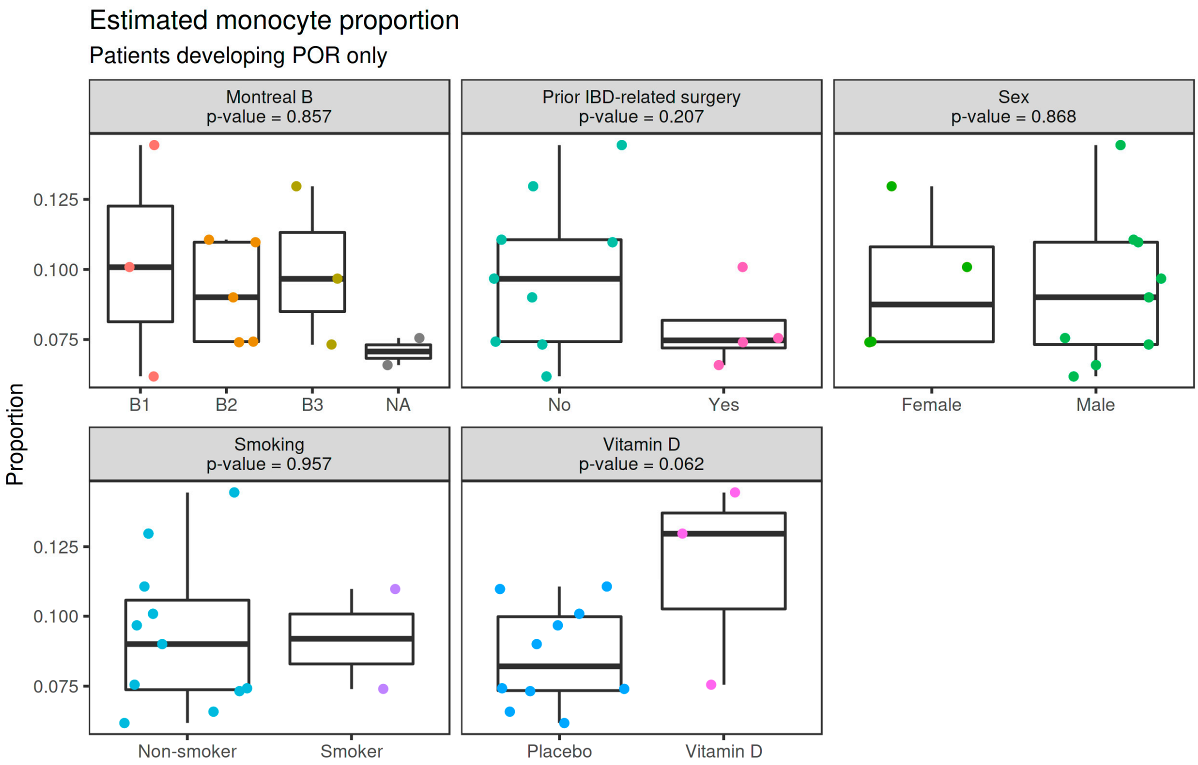

Supplementary Figure S1. Association of clinical parameters with estimated monocyte proportion. We estimated the blood cell distribution of the monocytes, NK-cells, CD8+ T-cells, B-cells, CD4+ T-cells and neutrophils using the IDOL dataset. The x-axis of each box indicates the difference between patients that remain in endoscopic remission (orange) vs. POR (blue), with corresponding p-values as—calculated using two-sample *t*-tests—shown above. The y-axis of each box shows the proportion of that particular cell type. A significantly higher proportion of monocytes at baseline were observed for patients that ultimately developed POR.
